# IMP PCR primers detect single nucleotide polymorphisms for *Anopheles gambiae *species identification, Mopti and Savanna rDNA types, and resistance to dieldrin in *Anopheles arabiensis*

**DOI:** 10.1186/1475-2875-5-125

**Published:** 2006-12-19

**Authors:** Elien E Wilkins, Paul I Howell, Mark Q Benedict

**Affiliations:** 1 Atlanta Research & Education Foundation (AREF), Atlanta, GA, USA; 2 Centers for Disease Control and Prevention (CDC), 4770 Buford Hwy. MS F-42, Atlanta, GA 30341. Phone 770-488-4987, FAX 770-488-4258, USA

## Abstract

**Background:**

Polymerase chain reactions to distinguish single-nucleotide polymorphisms are commonly used for mosquito identification and identifying insecticide resistance alleles. However, the existing methods used for primer design often result in analyses that are not robust or require additional steps.

**Methods:**

Utilizing oligonucleotides that are unique in having an intentional mismatch to both templates three bases from the SNP at the 3-prime end, three new PCR assays that distinguish SNP targets using standard gel electrophoresis of undigested DNA fragments were developed and tested. These were applied to: (1) an alternative ribosomal DNA PCR assay to distinguish five members of the *Anopheles gambiae *complex; (2) detection of the Mopti and Savanna rDNA types; and (3) an assay to distinguish *resistance to dieldrin *(*Rdl*) alleles in *Anopheles arabiensis*.

**Results:**

Reproducible specific amplification of the target alleles was observed in all three assays. The results were consistent with existing analyses but proved simpler and the results more distinct in our hands.

**Conclusion:**

The simplicity and effectiveness of the method should be utilized in these and other PCR analyses to increase their specificity and simplicity. These results have the potential to be extended not only to mosquito analyses but also to parasite and human polymorphisms.

## Background

Single nucleotide polymorphisms (SNPs) have been utilized as markers for species differentiation [1] and are implicated in insecticide resistance in many anopheline mosquitoes [2]. While various polymerase chain reaction (PCR) approaches to discriminate SNPs exist, specific primers for the wild-type and/or mutant alleles are often designed to perfectly match the sequence of one allele whereas the second varies only by matching the alternative SNP sequence at the terminal 3' end. Therefore, it is critical that the biochemical specificity of the two primers is sufficient to determine the success of PCR amplification from a particular template. This PCR-specificity depends on two characteristics: the ability of any mismatch to prevent extension of the primer by the polymerase – many polymerases can synthesize template over a single terminal mismatch [3] – and the difference between the annealing temperature (T_m_) of the perfect and mismatched primer [4]. For both of these reasons, a single 3' mismatch has limited discrimination. In this report, intentional mismatches were used to increase the discrimination of PCR based primarily on the polymerase synthesis characteristic.

Three PCR analyses were created by the method described here:

(1) The *Anopheles gambiae *complex is comprised of six genetically and behaviourally distinct species that are morphologically almost identical [5]. Members of this complex have been identified as major vectors of human malaria parasites, and as many as four species may be sympatric. Several methods for identifying these species have been developed, such as gas chromatography of cuticular hydrocarbons [6], polytene chromosome arrangements (reviewed in [7]) and allozyme analyses [8]. However, none of these has obtained as widespread use as PCR amplification of the ribosomal DNA (rDNA). This has become the standard method for species discrimination since rDNA polymorphism detected by Southern hybridization [9] spurred development of a PCR assay [1]. While the Scott et al. PCR method is the most popular, non-specific bands and high rates of PCR amplification failure were observed in our hands.

(2) In addition to the cryptic species of the *An. gambiae *complex described above, karyotype analysis of the female ovarian chromosomes has revealed further subdivision into chromosomal forms called Forrest, Mopti (M) and Savanna (S) [7]. The observed frequencies of the inversions that define these types are correlated with different ecological zones suggesting that they may have an adaptive character. Because karyotype analysis is tedious and can only be performed on semi-gravid females, several alternative PCR-based methods have been developed. PCR amplification of the rDNA intergenic spacer IGS [10-12] and internal transcribed spacers ITS1 and ITS2 [13] of *An. gambiae s.s*. have provided a molecular surrogate for karyotypes of Mopti and Savanna types in some but not all areas [13]. The novel method reported here was compared with the diagnostic PCR-RFLP method of Fanello [11] which amplifies a portion of the IGS followed by digestion of the products with *Hha *I to produce diagnostic fragments of either rDNA type.

(3) *Resistance to dieldrin *(*Rdl*) in *An. gambiae s. l*. is due to a single nucleotide mutation within the M2 transmembrane subunit of γ-aminobutyric acid (GABA) receptor [14]. In *Anopheles arabiensis*, resistance is conferred by an alanine to serine substitution within the *Rdl *locus. Resistance to dieldrin *per se *(a cyclodiene) is of little immediate concern since it has been banned from use in public health and agriculture. However, recently it has been shown that cross-resistance to the phenylpyrazole fipronil in *Anopheles stephensi *and *An. gambiae *was also due to the presence of an *Rdl *resistance allele [15,16].

In this report, intentional mismatch primers – IMPs- a method previously utilized by Papp et al. [17] – were used to introduce mismatches at the third nucleotide from the 3' end. This simple modification of intentionally adding a mismatch to the SNP-detection primers counter-intuitively *increases *the specificity of the primers, which is demonstrated here for three anopheline SNP detection analyses.

## Methods

### Mosquitoes

Mosquitoes were obtained from the Malaria Research and Reference Reagent Resource Center (MR4) holdings at the CDC in Atlanta, GA USA (Table 1). Larvae were reared at 27°C using a standard method [18] except that larva were fed Aquaricare™ Koi Floating Blend from the L2 stage to pupation.

**Table 1:** Mosquito stocks

		Analysis				
				
Species	Stock	rDNA	Rd1	Species ID	MR4 no.	Origin
*An. gambiae s.s*	MOPTI	X			MRA-763	Mali
	KISUMU1	X		X	MRA-762	Kenya
*An. merus*	OPHANSI			X	MRA-801	South Africa
*An. quadriannulatus*	SKUQUA			X	MRA-761	South Africa
*An. arabiensis*	KGB		X	X	MRA-339	Zimbabwe
	SENN		X		MRA-764	Sudan

### PCR

Samples were prepared for PCR by the method of Rafferty et al. [19]. PCR products were observed by separation on 0.5× TBE agarose gels run in 0.5× TBE buffer at 12 v/cm and fragment sizes were estimated using a 1 kb ladder marker (Invitrogen^®^). Thermal cycling for all analyses was performed in a Bio-Rad iCycler^®^. *Taq *DNA polymerase and the manufacturer's (Promega^®^) recommended buffer at 1× concentration was used for all reactions. PCR reactions consisted of 1 U of *Taq *polymerase, 0.3 mM MgCl_2_, all primers at 1 μM except QD-3T at 2 μM, 0.08 mM dNTPs, and buffer in 25 μl total volume. All primers used in these studies except those of Scott et al. are listed in Table 2.

**Table 2:** Primer sequences. Lower case nucleotide indicates the intentional mismatch, nucleotides in bold are located at site of SNP (where applicable), F and R indicate forward and reverse orientation.

*An. gambiae *species ID			(5' to 3')Fragment (size bp)
IMP-UN:	F	GCTGCGAGTTGTAGAGATGCG	
	R	GCATGTCCACCAACGTAAAtC**C**	*An. quadriannulatus *(637)
QD-3T	R	CAACCCACTCCCTTGACGaT**G**	*An. melas *and *merus *(529)
ME-3T	R	GCTTACTGGTTTGGTCGGCAtG**T**	*An. gambiae *(464)
GA-3T AR-3T	R	GTGTTAAGTGTCCTTCTCCgT**C**	*An. arabiensis *(388)
Mopti/Savanna rDNA typing			
M5	F	CTTGGTCTGGAGACCGTTCCaT**A**	Mopti (426)
M3	R	GACACGTCAACTAAGTCAACACATtA**C**	
S5	F	GCCCCTTCCTCGATGGaG**C**	Savanna (335)
S3	R	CAACCGGCCCAAACGGcT**T**	
*Rdl *mutation assay			
RDLF	F	AGTTTGTACGTTCGATGGGTTA	positive control (256)
RDLR	R	CCAGCAGACTGGCAAATACC	
AARDL	F	GCTACACCAGCACGTGaT**T**	dieldrin resistant (158)
RDLSS	R	CAAGACAGTAGTTACACCTAAaG**C**	dieldrin susceptible (121)

*An. gambiae *species identification PCR cycling consisted of melting at 95°C for 5 min followed by 30 cycles of 95°C for 30 seconds, 58°C for 30 seconds, and 72°C for 30 seconds, followed by one cycle of 72°C for 5 min. Mopti and Savanna rDNA were analysed using PCR-amplified rDNA and *Hha *I digests by the method of Fanello [11]. *Hha *I digests were performed overnight at 37°C. The locations of novel primers were selected at SNP sites using published sequence data [Genbank: AF470116 and AF470112]. PCR thermal cycling for the IMP primers consisted of melting at 95°C for 5 min followed by 30 cycles of 95°C for 30 seconds, 54°C for 30 seconds, and 72°C for 30 seconds, followed by one cycle of 72°C for 5 min.

*An. arabiensis Rdl*-specific primers were designed to be used with the previously reported *An. gambiae *internal positive control primers RDLF and RDLR [14]. Thermal cycling consisted of melting at 95°C for 5 min followed by 30 amplification cycles of 95°C for 45 seconds, 53°C for 45 seconds, and 72°C for 45 seconds, followed by one cycle of 72°C for 10 min. Heterozygous F_1 _individuals were produced for analysis by crossing KGB (homozygous susceptible) females to SENN (homozygous resistant) males *en masse*.

## Results and discussion

**Figure 1:**
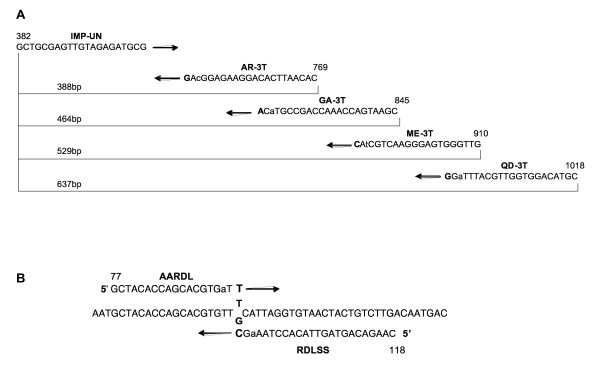
Schematic alignment of IMP primers. (A) *An. gambiae *identification rDNA primers. The bp numbering is that of Scott et al. (1993). SNP sites are in bold, lower case nucleotides are intentional mismatches. The IMP-UN primer is shown in 5' to 3' orientation, whereas the reverse complement of all reverse primers are shown. (B) Alignment of *Rdl *primers. The bp numbering is as previously designated [Genbank: AY787486]. SNP sites are in bold, lower case nucleotides are intentional mismatches. The SNP sequence is T or G in the resistant and susceptible alleles respectively.

**Figure 2:**
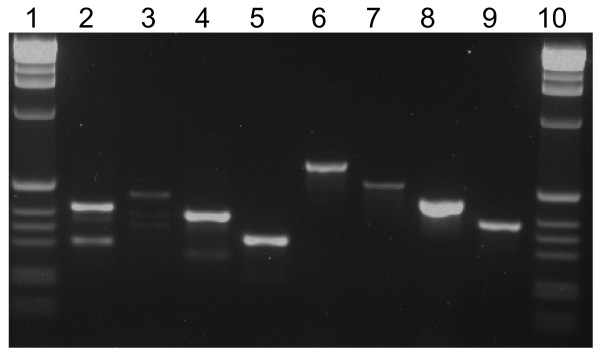
Gel electrophoresis of *An. gambiae *species identification comparing products produced by the method of Scott et al. and the IMP method presented in this paper. Lanes contain: (1, 10) 1 kb ladder marker, (2–5) Scott et al. method, (2) *An. quadriannulatus*, (3) *An. merus*, (4) *An. gambiae *(KISUMU1), (5) *An. arabiensis *(KGB), (6–9) IMP method, (6) *An. quadriannulatus*, (7) *An. merus*, (8) *An. gambiae *(KISUMU1), and (9) *An. arabiensis *(KGB).

The primer strategy for *An. gambiae *species identification was similar to that of Scott et al. [1]: one universal forward primer was used for all species in a cocktail with four species-specific reverse primers. However, neither the species-specific nor universal annealing sites were the same (Figure 1a). The species-specific DNA fragments that we obtain using the IMP primer set for *An. gambiae s.l*. species identification (Table 2) compare favorably to the products we obtain with the Scott et al. assay (Figure 2). While in the latter assay, non-specific artifact bands were observed, the IMP-protocol products are clear and consistent. Prior to developing these assays, primers were designed for the species identification except incorporating the deliberate mismatch at the penultimate base. These did not provide the desired level of specificity with the mismatch at this site. Additional primers were designed with a mismatch and the SNP in the middle in an attempt to thermodynamically alter the melting temperature sufficiently for discrimination. Neither of these approaches was successful.

In this study, only laboratory colonies of mosquitoes were tested for species ID, and validation on a larger scale as has been performed previously for *Anopheles quadrimaculatus s.l*. [20] will be necessary to confirm their utility. In order to ensure that the IMP primer sets would function correctly in strains other than those we analysed and for which sequence data was available, reported sequences were examined [1,12,21-23] and no polymorphisms were found that would interfere with the success of the protocol.

**Figure 3:**
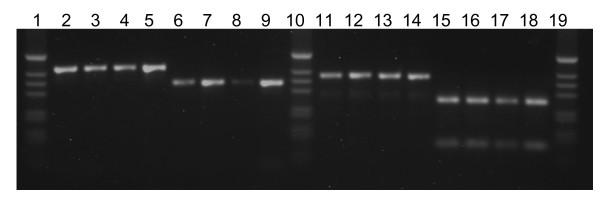
Gel electrophoresis of Mopti – Savanna rDNA assay. Lanes 2–5, 11–14 and 6–9, 15–18 contain Mopti and Savanna PCR products respectively. Lanes 2–9 were performed using the IMP primers and reactions. Lanes 11–18 were performed by the method of Fanello. Lanes 1, 10, and 19 contain 1 kb ladder marker.

An alternative to the Fanello protocol for analysis of the IGS was developed that is in principle like that of Favia et al. [12] in that PCR, but not restriction digestion, is required. In the Fanello analysis, the expected resulting fragments of the M (367 + 23 bp) and S (257 + 110 + 23 bp) types include one only slightly smaller than the undigested 390 bp PCR product, therefore, high percentage agarose gels are used to distinguish products. The IMP primer design includes single base mismatches between the M and S rDNA in the target regions. They were designed to amplify distinct fragments of 426 or 335 bp in the M and S forms respectively (Figure. 3). The results with these primers are consistent with those of the Fanello protocol and additional evidence of their consistency was obtained in a larger unrelated study.

**Figure 4:**
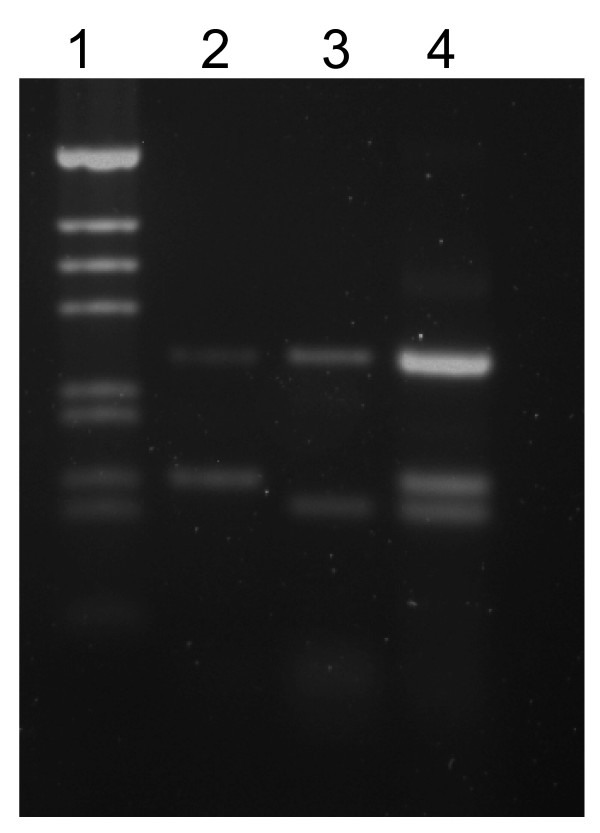
Gel electrophoresis of *resistance to dieldrin *assay. Lanes contain: (1) 1 kb ladder marker, (2) *An. arabiensis *dieldrin resistant (SENN), (3) *An. arabiensis *dieldrin susceptible (KGB), (4) *An. arabiensis *heterozygote from SENN × KGB.

The *Rdl *dieldrin resistance PCR assay contains two perfect-match primers (RDLF & RDLR, [14]) that amplify the region containing the SNP and one forward and one reverse IMP primer that specifically bind the SNP found in *An. arabiensis*. The SNP site in *An. arabiensis *is located asymmetrically from the middle of the region between the RDLF and RDLR primers so we were able to design a forward and a reverse primer at that site (Figure 1b), the forward primer being specific for the resistant form and the reverse specific for the susceptible form. The four primers are used in a cocktail together and are expected to yield a control band and a resistant or susceptible band in homozygotes depending on the templates. The result of using the four-primer cocktail with a heterozygote yielded three bands as expected (Figure 4).

The novel method, upon which these assays is based, is that all SNP-specific primers were designed to contain an intentional mismatch for both target alleles at the third nucleotide from the 3' end and with the terminal base falling on the site of the SNP [17]. These primers were tested over a wide range of annealing temperatures using a gradient cycler and were found to amplify their targets consistently and clearly over annealing temperatures of 50–65°C. This bodes favorably for reproducibility of the results obtained here on different machines. Successful amplification over a wide range of temperatures also suggests that the primer design is successful not due to a thermodynamic change leading to a lower T_m _but rather due to the inability of a standard Taq polymerase to extend over a series with alternating mismatches at the 3' end.

## Conclusion

Successful amplification using IMPs was achieved with all mosquito stages tested including larvae, pupae, and adult mosquitoes without extensive DNA purification. Therefore, these assays are useful for studies in the field where DNA preparation is not possible or time-prohibitive. The ease of design, technical simplicity, high specificity, and sensitivity recommend these as improved specific assays and the general method as a means to produce assays for both multicopy and single-copy SNPs for laboratory and field analyses. There is no apparent reason that similar primer designs would not be useful for analysis of human and *Plasmodium *SNPs and any application in which rapid sensitive SNP discrimination is necessary.

## Authors' contributions

EW carried out the primer design research, primer design and performed the first draft of the manuscript. PH participated in background research. Both EW and PH conducted molecular analysis, mosquito culture, and reviewed numerous revisions of the manuscript. MB participated in the design and coordination of the studies and helped to draft and performed the final review of the manuscript. All authors read and approved the final manuscript.
